# Exploring the relationship of static and dynamic balance with muscle mechanical properties of the lower limbs in healthy young adults

**DOI:** 10.3389/fphys.2023.1168314

**Published:** 2023-05-26

**Authors:** Mathew W. Hill, Maximilian M. Wdowski, Katarzyna Rosicka, Anthony D. Kay, Thomas Muehlbauer

**Affiliations:** ^1^ Centre for Sport, Exercise and Life Sciences, School of Life Sciences, Coventry University, Coventry, United Kingdom; ^2^ Department of Physiotherapy Faculty of Physical Culture in Gorzów Wlkp, Poznań University of Physical Education, Gorzów Wlkp, Poland; ^3^ Centre for Physical Activity and Life Sciences, Faculty of Art, Science and Technology, University of Northampton, Northamptonshire, United Kingdom; ^4^ Division of Movement and Training Sciences/Biomechanics of Sport, University of Duisburg-Essen, Essen, Germany

**Keywords:** myotonometry, biomechanical properties, muscle stiffness, viscoelasticity, postural control, centre of pressure displacement, distance reaching

## Abstract

There is emerging evidence that mechanical properties of *in vivo* muscle tissues are associated with postural sway during quiet standing. However, it is unknown if the observed relationship between mechanical properties with static balance parameters generalise to dynamic balance. Thus, we determined the relationship between static and dynamic balance parameters with muscle mechanical properties of the ankle plantar flexors [lateral gastrocnemius (GL)] and knee extensors [vastus lateralis (VL)] *in vivo*. Twenty-six participants (men = 16, women = 10; age = 23.3 ± 4.4 years) were assessed for static balance [centre of pressure (COP) movements during quiet standing], dynamic balance (reach distances for the Y-balance test) and mechanical properties (stiffness and tone) of the GL and VL measured in the standing and lying position. Significant (*p* < .05) small to moderate inverse correlations were observed between the mean COP velocity during quiet standing with stiffness (*r* = −.40 to −.58, *p* = .002 to .042) and tone (*r* = −0.42 to −0.56, *p* = 0.003 to 0.036) of the GL and VL (lying and standing). Tone and stiffness explained 16%–33% of the variance in the mean COP velocity. Stiffness and tone of the VL measured in the lying (supine) condition were also inversely significantly correlated with Y balance test performance (r = −0.39 to −0.46, p = 0.018 to 0.049). These findings highlight that individuals with low muscle stiffness and tone exhibit faster COP movements during quiet standing, indicative of reduced postural control but also reveal that low VL stiffness and tone are associated with greater reach distances in a lower extremity reaching task, indicative of greater neuromuscular performance.

## 1 Introduction

It is firmly established that a considerable proportion of postural sway is controlled at the ankle joint during static balance conditions (i.e., quiet standing) (Gatev et al., 1999), with ankle torque generated via passive and active mechanisms (Sakanaka et al., 2021). Quiet standing demands active neural control via modulation of muscle activity signalled by the central nervous system (Sakanaka et al., 2018) because the passive compensation mechanisms are insufficient to stabilise the body during upright stance (Loram and Lakie, 2002; Casadio et al., 2005). Whilst active feedback control is delayed by about 190–210 ms (Peterka, 2002), passive mechanisms produce viscoelastic forces with zero delay (Sakanaka et al., 2018), allowing more time for the nervous system to provide an active response (Sakanaka et al., 2021). As the instantaneous torque provided by passive control mechanisms play an important role in maintaining upright stance, understanding how mechanical properties of the musculoskeletal system relate to balance performance is of significant value to researchers and clinicians.

A variety of techniques exist for non-invasively quantifying mechanical properties of soft myofascial tissues *in vivo*, including shear wave elastography, tensiomyography and myotonometry, each with their own advantages and disadvantages. Myotonometry has proven a promising technique that provides a reliable assessment of stiffness in postural muscles (Bizzini and Mannion, 2003; Feng et al., 2018; Chen et al., 2019) whilst requiring less technical expertise than elastography measurements (Bravo-Sanchez et al., 2021). Recent findings indicate that mechanical properties of muscle tissues derived from tensiomyography (Shin et al., 2019) and myotonometry (Vain et al., 2015) are associated with static balance parameters [centre of pressure (COP) displacements]. Although the findings of Shin et al. (2019) and Vain et al. (2015) imply that postural control during quiet stance is, in part, related to intrinsic muscle stiffness in the lower extremities, one limitation of this research is that the findings are only generalisable to maintenance of static balance, with the relationship between muscle mechanical properties and dynamic balance yet to be established.

Static and dynamic balance tasks represent distinct qualities that are not interchangeable and are reliant upon different control mechanisms (Kiss et al., 2018; Ringhof and Stein, 2018). As such, it cannot be assumed that the observed relationship between mechanical properties with static balance parameters will generalise to dynamic balance. Furthermore, young adults mainly use muscles encompassing the ankle joint to minimise postural sway during quiet standing (Donath et al., 2016) with the ankle muscles but primarily the knee extensors more important contributors during dynamic lower extremity reaching tasks (Hoch et al., 2011; Norris and Trudelle-Jackson, 2011). Given the information above, the present work aimed to scrutinise the relationship between muscle mechanical properties of the ankle plantar flexors [lateral gastrocnemius (GL)] and knee extensors [vastus lateralis (VL)] with static (COP displacements during quiet standing) and dynamic (reach distances for the Y-balance test) balance parameters, separately. Although exploratory, we hypothesised that GL and VL muscle mechanical properties (stiffness and tone) would be inversely associated with static and dynamic balance parameters.

## 2 Methods

### 2.1 Participants and sample size estimation

Previous studies have reported large magnitude associations between mechanical properties and postural sway parameters (Vain et al., 2015; Shin et al., 2019). Power analysis (G*Power, v3.1.9.4) showed that for a Pearson’s correlation analysis a minimum of 23 participants would be required to detect a significant moderate magnitude correlation (assuming 1-*β* = 80%, *α* = .05, *r* = .55). Twenty-six healthy young adults (men = 16, women = 10, age = 23.3 ± 4.4 years, body height = 1.74 ± 0.08 m, body mass = 71.5 ± 14.1 kg, BMI = 23.4 ± 3.3 kg/m^2^) volunteered to take part in the study. All participants self-reported as being recreationally active (IPAQ = 2.2 ± 1.8 h wk^-1^) and completed a health screening questionnaire to assess eligibility for the study and were free from any musculoskeletal injury or history of injury in the previous 6 months. Prior to data collection, the study received approval by the institutional ethics committee (approval number: P109131) with participants were providing written, informed consent and the experimental procedures were carried out in accordance with the Declaration of Helsinki (1964).

### 2.2 Assessment of muscle mechanical properties

Muscle mechanical properties of the GL and VL were measured using a hand-held myotonometer (Myoton-Pro, Myoton AS, Tallinn, Estonia). Participants were asked to rest for 10 minutes before assessment with all measurements taken on the dominant side (determined as the foot used to kick a ball), under two conditions: 1) lying on an assessment table in a prone (for GL) and supine (for VL) position with the feet hanging off the table at an ankle angle of 90°, and 2) standing quietly with the feet together. The lying and standing positions were employed to represent relaxed and semi-contracted states, respectively. The lying position examination was always performed first. Measurement regions were marked by the same experienced examiner to eliminate inter-tester variability. The measurement sites were standardised for all participants and established according to previous studies; VL was measured at 50% of the straight-line distance between the greater trochanter and fibulae capitulum (Bizzini and Mannion, 2003) and the GL was measured at 30% of the distance between fibulae capitulum and Achilles tendon insertion (Feng et al., 2018). The probe of the myotonometer was placed perpendicular to the surface of the skin at both measurement sites and applied a brief mechanical compression (0.4 N for 15 ms) with a constant preload force of 0.18 N. The myotonometer accelerometer was set at 3,200 Hz with an average value obtained from five consecutive measurements. Each site was measured three times and an average of those measures were used in the subsequent analyses. The oscillation acceleration signal allows for the calculation of mechanical properties of stiffness (N∙m^-1^) (i.e., ability to resist an external force that modified its shape) and non-neural tension or tone (frequency; Hz) (i.e., intrinsic tension on a cellular level without voluntary contraction). Test-retest reliability revealed ‘excellent’ ICC-values for the VL in the standing (stiffness: ICC = .987–.989, tone: ICC = .984–.988) and lying (stiffness: ICC = .961–.978, tone: ICC = .975–.986) condition. For the GL, we also obtained ‘excellent’ ICC-values in the standing (stiffness: ICC = .985–.993, tone: ICC = .982–.986) and lying (stiffness: ICC = .984–.992, tone: ICC = .981–.988) condition.

### 2.3 Assessment of static balance

Two minutes after the assessment of muscle mechanical properties, participants performed three 30-s quiet standing trials on a force platform (AMTI, AccuGait, Watertown, MA) with the eyes open. Participants were unshod with the hands clasped together in front of the body and with the feet together (Objero et al., 2019). During all trials, participants were asked to stand still while gazing at a black circle 1.5 m away from the force platform, adjusted to the eye level of each individual. Ground reaction force data were sampled at 100 Hz (Netforce, AMTI, Watertown, MA) and filtered using a fourth-order low-pass (6 Hz) Butterworth filter (BioAnalysis V2.2, AMTI, Watertown, MA) prior to calculation of COP metrics. The maximal range of the COP in the anteroposterior (AP) and mediolateral (ML) directions (cm) and the mean COP velocity (cm∙s^-1^) were subsequently calculated (BioAnalysis V2.2). The AP and ML ranges express the distance between the most distal points of the COP displacement in the frontal (ML) and sagittal (AP) planes, whilst the mean COP velocity is obtained by dividing the total distance travelled (i.e., COP path length) by the sampling duration (Roman-Liu, 2018). An average of the three trials were used in subsequent analyses (Pinsault and Vuillerme, 2009).

### 2.4 Assessment of dynamic balance

Two minutes following the assessment of static balance, participants completed the dynamic balance assessment using the Y Balance Test Kit™. While maintaining a single-leg stance with the dominant limb, participants were asked to push a reach indicator along a pipe with the contralateral limb (i.e., non-dominant limb) in a randomised order in the anterior (ANT), posteromedial (PM), and posterolateral (PL) reach directions. Participants were instructed to place the arms akimbo to remove the potential effects of arm movements on postural control (Objero et al., 2019). The test was discarded and repeated if the participant 1) failed to maintain single-leg stance (i.e., touched the floor with the reach limb), 2) failed to remain in contact with the reach indicator at the most distal point (i.e., kicked the reach indicator to achieve greater distance), 3) used the reach indicator to support weight (i.e., mechanical support), 4) failed to return the reach foot to the centre of the foot plate, or 5) failed to keep their arms akimbo. Participants performed three trials for each reach direction with the greatest reach distance in each direction used for subsequent analysis. Reach distance was normalised to dominant limb length (reach distance/limb length * 100) (Plisky et al., 2006) measured in centimetres (cm) from the anterior superior iliac spine to the most distal portion of the medial malleolus using anthropometric measuring tape (Gribble and Hertel, 2003). The composite (COMP) reach score was calculated as the sum of the three reach directions divided by limb length, and then multiplied by 100 (Plisky et al., 2006).

### 2.5 Statistical analysis

Data were analysed using SPSS version 26.0 (IBM Inc., Chicago, IL) and are presented as mean ± standard deviation (SD) and range. After normal distribution was confirmed (Shapiro-Wilk tests), associations between muscle mechanical properties with COP parameters and normalised reach distances were separately assessed using Pearson’s product moment correlation coefficient. Values were interpreted as weak (*r* = .10 to .35), moderate (*r* = .36 to .67), or strong (*r* = .68 to 1.00) (Taylor, 1990). The alpha value was *a priori* set at *p* < .05 for all analyses. Further, the amount of variance explained was reported by the coefficient of determination (*r* ^2^).

## 3 Results

Table 1 shows the participants characteristics, the muscle mechanical properties, and the dynamic as well as static balance outcomes.

**TABLE 1:** Means ± standard deviation (SD) for the demographic and performance characteristics of the sample (*N* = 26).

Dependent variable	Mean ± SD	Range
Demographics and functional status		
Age (years)	23.3 ± 4.4	18–32
Body height m)	1.7 ± 0.1	1.6–1.9
Body mass (kg)	71.5 ± 14.1	51–98
Leg length (cm)	92 ± 4.9	83–99
BMI (kg/m^2^)	23.4 ± 3.3	18.3–30.2
IPAQ (h∙wk^-1^)	3.5 ± 2.1	1–7
**VL Mechanical Properties (lying, supine)**		
Stiffness (N/m)	311.9 ± 41.9	249.7–400.7
Tone (Hz)	16.6 ± 2.1	13.5–20.8
**GL Mechanical Properties (lying, prone)**		
Stiffness (N/m)	313.0 ± 59.6	207.3–463.3
Tone (Hz)	17.4 ± 2.4	13.0–21.9
**VL Mechanical Properties (standing)**		
Stiffness (N/m)	394.6 ± 100.1	229.0–601.3
Tone (Hz)	18.4 ± 3.3	12.8–24.8
**GL Mechanical Properties (standing)**		
Stiffness (N/m)	403.3 ± 96.7	228.0–571.7
Tone (Hz)	19.6 ± 3.3	13.9–24.9
**Static balance**		
AP range (cm)	2.0 ± 0.3	1.4–2.7
ML range (cm)	1.9 ± 0.3	1.3–2.5
Mean COP velocity (cm∙s^-1^)	2.4 ± 0.4	1.8–3.0
**Dynamic balance**		
ANT (% LL)	74.0 ± 9.3	62.8–94.9
PL (% LL)	111.8 ± 12.4	87.5–134.7
PM (% LL)	115.2 ± 11.3	94.5–138.6
COMP (% LL)	109.7 ± 15.3	84.7–142.3

Note. ANT, anterior reach distance; AP, anteroposterior centre-of-pressure range; BMI, body mass index; COMP, composite reach score; GL, gastrocnemius lateralis; IPAQ, international physical activity questionnaire; LL, leg length; ML, mediolateral centre-of-pressure range; PL, posterolateral reach distance; PM, posteromedial reach distance; VL, vastus lateralis.

### 3.1 Correlations between muscle mechanical properties and static balance

**FIGURE 1:**
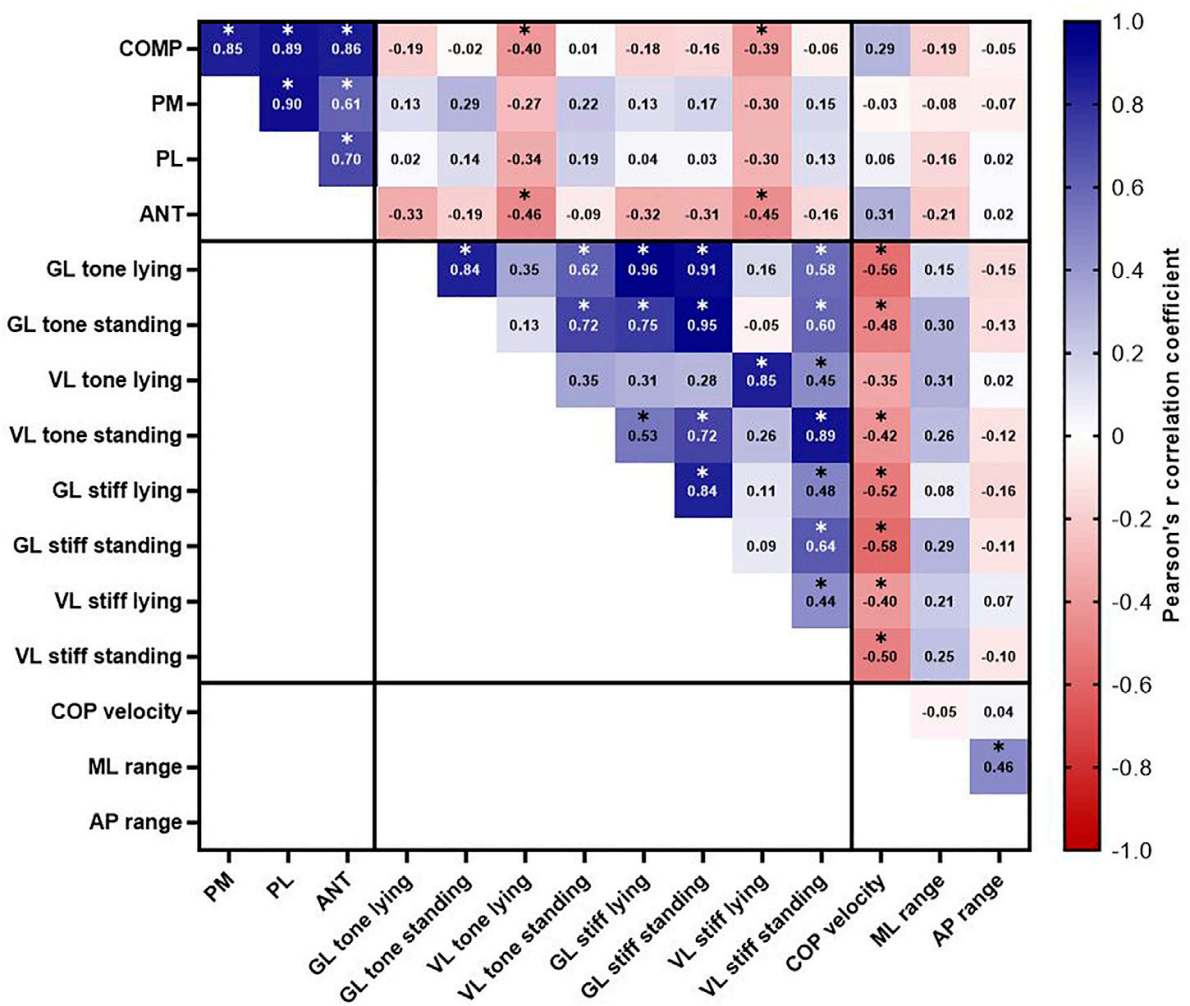
Correlation matrix for the associations between muscle mechanical properties with static and dynamic balance where values indicate Pearson’s *r* correlation coefficient. *Indicates statistically significant correlation (*p* < .05). Shading indicates strength of relationship (blue = positive correlation, red = negative correlation). Note. ANT, anterior reach distance; AP, anteroposterior COP range; COMP, composite reach score; COP, centre of pressure; GL, lateral gastrocnemius; ML, mediolateral COP range; PL, Posterolateral reach distance; PM, posteromedial reach distance; Stiff, stiffness; VL, vastus lateralis.

Associations between muscle mechanical properties with static and dynamic balance parameters were examined by Pearson’s correlation matrix (Figure 1).

**FIGURE 2:**
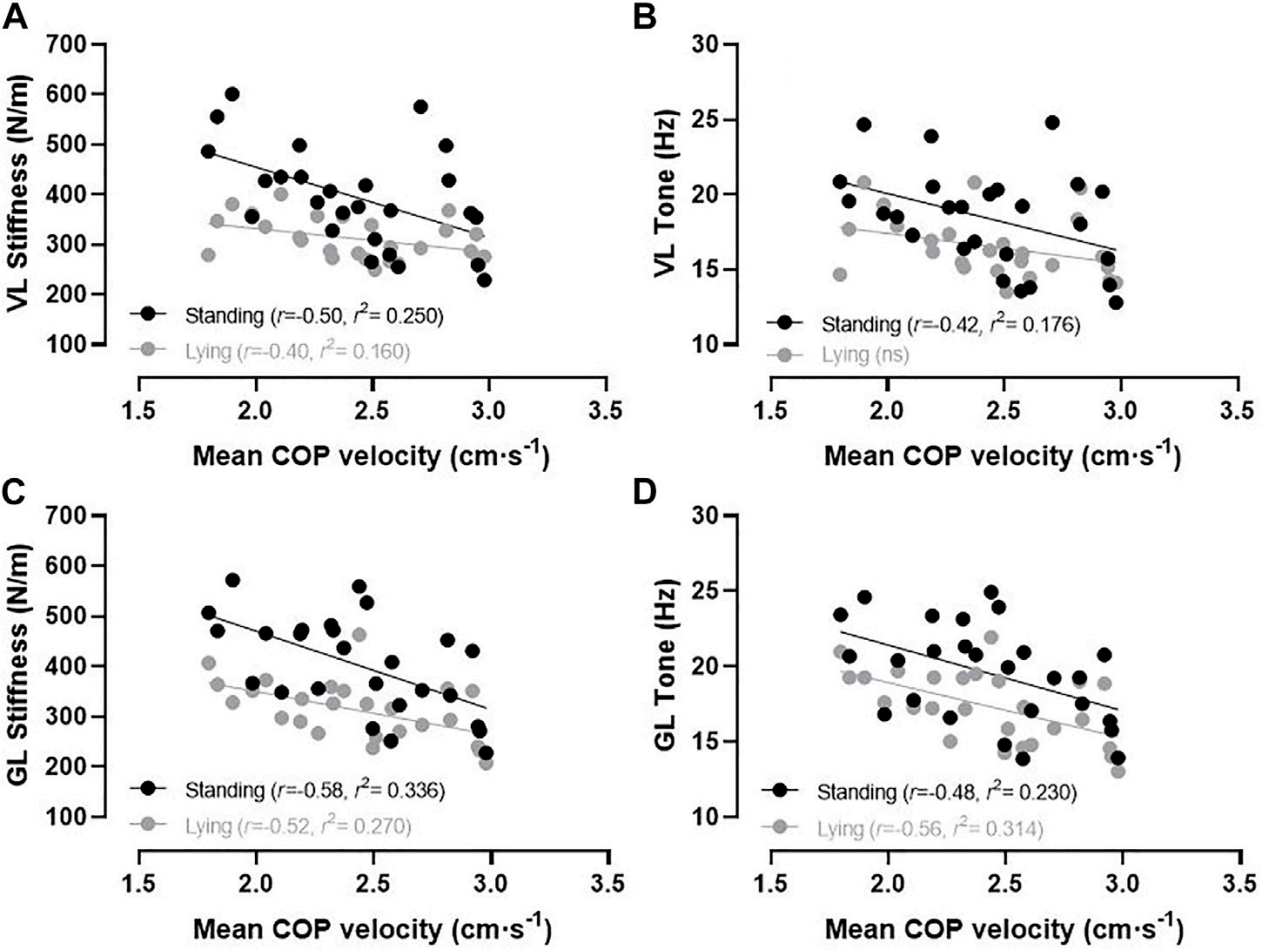
Relationships between VL stiffness **(A)**, VL tone **(B)**, GL stiffness **(C)**, and GL tone **(D)** during standing (black circles) and lying (grey circles) conditions with the mean COP velocity during quiet standing. Note. COP, centre of pressure; GL, lateral gastrocnemius; ns = not statistically significant; *r*, correlation coefficient; *r* ^2^, coefficient of determination; VL, vastus lateralis.

The analyses revealed significant moderate magnitude inverse correlations for VL (*p* = .009, *r* = −.50) and GL (*p* = .002, *r* = −.58) stiffness measured in the standing condition with the mean COP velocity. Similarly, statistically significant small inverse correlations were observed for VL (*p* = .036, *r* = −.42) and GL (*p* = .015, *r* = −.48) tone measured in the standing condition with the mean COP velocity. During the lying condition, GL tone (*p* = .003, *r* = −.56) and stiffness (*p* = .007, *r* = −.52) and VL tone (*p* > .05, *r* = −.35) and stiffness (*p* = .042, *r* = −.40) were small to moderately inversely associated with the mean COP velocity. Muscle mechanical properties of the GL and VL explained 16%–33% of the variance (see *r* ^2^-values) in the mean COP velocity during quiet standing (Figures 2A–D). The COP displacements in the AP and ML directions were not significantly correlated with any muscle mechanical properties (*p* > .05).

### 3.2 Correlations between muscle mechanical properties and dynamic balance

**FIGURE 3:**
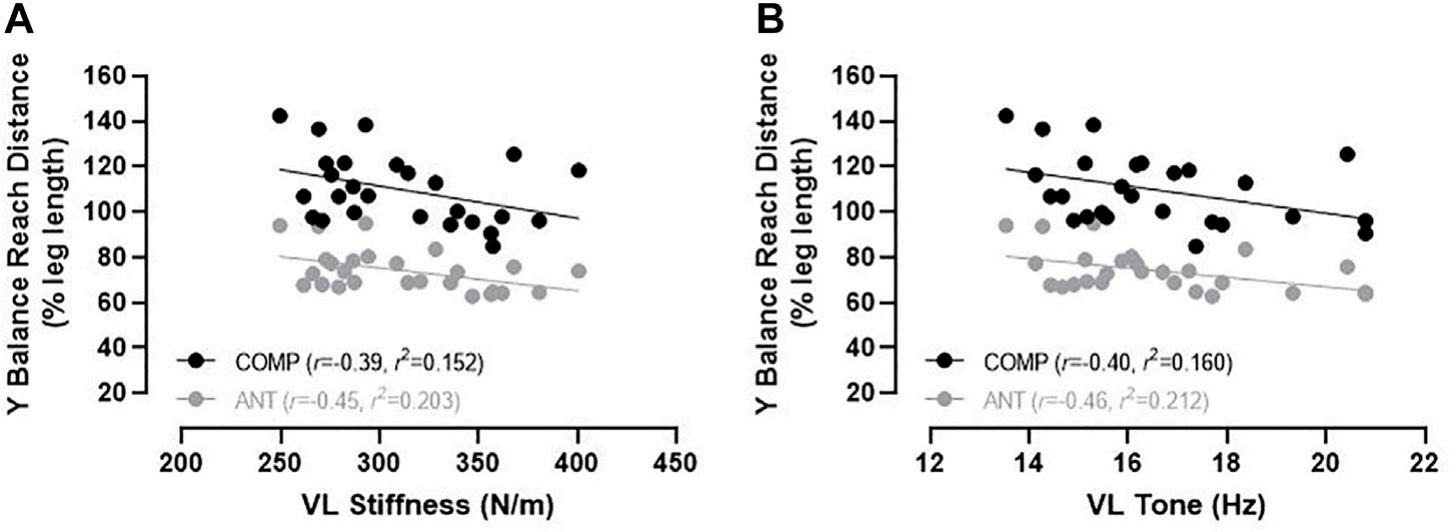
Relationship between VL stiffness **(A)** and VL tone **(B)** measured in the lying (supine) condition with the composite reach score (black circles) and the anterior reach distance (grey circles) for the Y Balance Test. Note. ANT, anterior reach distance; COMP; composite reach score; *r*, correlation coefficient; *r* ^2^, coefficient of determination; VL, vastus lateralis.

Most of the correlation coefficients between muscle mechanical properties and dynamic balance performance (28 out of 32 cases; Figure 1) were not statistically significant, however significant small magnitude inverse correlations between COMP reach performance with VL tone (*p* = .040, *r* = −.40) and VL stiffness (*p* = .049, *r* = −.39) were detected when measured in the lying (supine) condition. Similarly, ANT reach distance was inversely associated with VL tone (*p* = .018, *r* = −.46) and VL stiffness (*p* = .020, *r* = −.45) when measured in the lying (supine) condition. Muscle stiffness and tone of the VL when measured in the lying (supine) condition explained 15%–21% of the variance (see *r* ^2^-values) in ANT and COMP Y Balance Test performance (Figures 3A–B).

## 4 Discussion

In the present study, we explored the relationship between muscle mechanical properties of *in vivo* myofascial tissues with static and dynamic balance parameters, separately. Two novel findings emerged: First, GL and VL stiffness and tone measured in both standing and lying conditions were inversely correlated with the velocity (i.e., mean COP velocity), but not the amplitude (i.e., AP and ML range), of the COP during quiet standing (static balance). Second, VL stiffness and tone measured in the lying (supine) condition were inversely correlated with ANT and COMP reach distance (dynamic balance). These findings highlight that individuals with low muscle stiffness and tone exhibit faster COP movements during quiet standing, indicative of reduced postural control, but also reveal that low VL stiffness and tone are associated with greater reach distances in a lower extremity reaching task, indicative of greater dynamic balance performance.

### 4.1 Static balance and muscle mechanical properties

Although there is emerging evidence linking passive muscle mechanical properties with static balance performance (Vain et al., 2015; Shin et al., 2019), a unique observation in the present study was that GL and VL tone and stiffness (in 5 out of 6 cases) were inversely associated with the velocity, but not the amplitude, of COP movements (i.e., AP and ML range) during quiet stance. In other words, individuals with lower muscle stiffness and tone presented a faster sway but not greater sway amplitudes. Critically, tone and stiffness explained 16%–33% of the variance in the mean COP velocity, highlighting the importance of these properties to static postural control.

The distinct correlations between muscle mechanical properties with the velocity, but not the amplitude, of COP movements during quiet standing may be explained by the different types of information provided by these COP measures regarding postural performance and stability. For example, the range of COP movements in the AP and ML direction reflects the precision (or effectiveness) achieved by the postural control system (Prieto et al., 1996), whilst the mean COP velocity is thought to characterise the net neuromuscular activity (or efficiency) required to maintain an upright stance (Maki et al., 1990; Caron et al., 2000). From a feedback control model perspective, the inverse association between muscle mechanical properties and postural sway seems logical, since low muscle stiffness/tone could be expected to reduce the rate of force transmission (Behm et al., 2004) and increase muscle reaction time (Longo et al., 2017), where even small delays in the feedback loop could negatively influence the velocity of COP movements during quiet standing. Furthermore, a lower muscle tone/stiffness implies a lower gain to the muscle spindle, and therefore a reduced sensitivity to stretch (Needle et al., 2014). Consequently, reduced muscle-spindle sensitivity emanating from low muscle stiffness could negatively influence joint position sense (Proske and Gandevia, 2012), which is critical for quiet standing balance control (Lord et al., 1991). Collectively, these findings indicate that higher GL and VL muscle stiffness and tone may allow for quicker muscle responses and corrections to postural sway and lead to more efficient postural control during quiet standing.

### 4.2 Dynamic balance and muscle mechanical properties

The relationships between lower extremity reaching performance with muscle tone and stiffness were examined in the present study which is, to our knowledge, the first to directly examine the interdependence between dynamic balance performance and muscle mechanical properties. Given the critical involvement of the knee extensors during lower extremity reaching tasks (Earl and Hertel, 2001; Norris and Trudelle-Jackson, 2011), we initially hypothesised that a high VL stiffness and tone would be associated with better dynamic balance performance. Although most correlations between muscle properties with dynamic balance parameters were not statistically significant (28 out of 32 cases), contrary to expectation, higher VL tone and stiffness measured in the lying (supine) position (i.e., independent of CNS contribution and ligament/joint stiffness contributions) were associated with poorer ANT and COMP reach scores. At first this finding may seem counter-intuitive as a higher muscle stiffness could be expected to provide a greater resistive capacity against external loading (Blackburn et al., 2004; Secomb et al., 2015). One possibility is that increased peripheral passive muscle tone may cause fascial rigidity, which can hyper-stimulate the muscle spindle (Needle et al., 2014) and subsequently reduce muscle compliance (Stecco et al., 2014). Furthermore, excessive muscle tone could increase antagonist muscle tone (i.e., disrupt the agonist/antagonist relationship), which may limit joint range of motion (ROM) and mobility (Needle et al., 2014; Iwamoto et al., 2017). This is important because knee flexion (Robinson and Gribble, 2008) and ankle dorsiflexion ROM (Hoch et al., 2011) significantly influence dynamic balance score (SEBT anterior reach distance). Therefore, our findings highlight that the lower stiffness of the VL may contribute to greater knee joint ROM resulting in a greater dynamic performance score. However, we failed to detect any association between ankle muscle mechanical properties tone and stiffness (which are closely related to ROM) of the GL and dynamic balance performance. This is contrary to the above-mentioned study of Hoch et al. (2011) who showed significant corrections between ankle dorsiflexion ROM and dynamic balance measures (i.e., SEBT anterior reach distance). The reason for the discrepancy between our results and the findings reported by Hoch and colleagues may be the result of the differing methods applied for the assessment of muscle function. Specifically, we used a hand-held-dynamometer to investigate muscle mechanical properties, but Hoch et al. (2011) measured dorsiflexion in cm while performing the weight-bearing lunge test. Therefore, both methods should be combined in future studies in order to be able to carry out complementary analyses.

### 4.3 Limitations

This exploratory study provides a novel contribution to the literature and a solid platform for future investigations to scrutinise relationship between muscle mechanical properties with static and dynamic balance. However, the findings of the present study should be interpreted in light of the study limitations. Firstly, only the GL and VL muscles on the dominant side were assessed and therefore, our findings should not be generalised to other muscles within these muscle groups or to the contralateral limb. Additional examinations of other ankle (e.g., dorsiflexors) and hip (e.g., abductor-adductor) muscles and the Achilles tendon would be quite valuable given their importance to postural control. Secondly, the study was based on a relatively small convenience sample of 26 healthy young adults and whilst the homogeneity of our sample may have limited the influence of confounding demographic variables, the external validity of this study’s findings to broader groups, such as the older adults, is limited. This is especially important when we consider that older adults often change from an ankle to a hip strategy to control postural sway (Woollacott, 2000). Future research scrutinising the relationship between muscle mechanical properties and balance performance in older groups would be quite valuable.

## 5 Conclusion

This work provides additional insights into how muscle mechanical properties of *in vivo* muscle tissues are separately related to static and dynamic balance tasks. For the first time, our results highlight that muscle mechanical properties of the GL and VL are uniquely correlated with velocity but not magnitude indices of postural sway (static balance). More specifically, high muscle GL and VL tone and stiffness is associated with a more “efficient” postural control during quiet standing. We further found that muscle mechanical properties of the VL tone and stiffness are associated with anterior dynamic reach distance (dynamic balance). Future work should explore the relationship between muscle mechanical properties and postural control in more challenging task situations (i.e., visual and stance width manipulation) and in different populations (e.g., older adults and people with balance problems).

## Data Availability

The raw data supporting the conclusion of this article will be made available by the authors, without undue reservation.

## Abbreviations

ANT, Anterior reach distance; AP, Anteroposterior centre-of-pressure range; BMI, Body mass index; COMP, Composite reach score; COP, centre-of-pressure; GL, Gastrocnemius lateralis; IPAQ, International Physical Activity Questionnaire; ML, Mediolateral centre-of-pressure range; PL, Posterolateral reach distance; PM, Posteromedial reach distance; Stiff, stiffness; VL, vastus lateralis.
